# Viral Transmission Dynamics at Single-Cell Resolution Reveal Transiently Immune Subpopulations Caused by a Carrier State Association

**DOI:** 10.1371/journal.pgen.1005770

**Published:** 2015-12-31

**Authors:** William Cenens, Angela Makumi, Sander K. Govers, Rob Lavigne, Abram Aertsen

**Affiliations:** 1 Laboratory of Food Microbiology, Department of Microbial and Molecular Systems (M²S), Faculty of Bioscience Engineering, KU Leuven, Leuven, Belgium; 2 Laboratory of Gene Technology, Department of Biosystems, Faculty of Bioscience Engineering, KU Leuven, Leuven, Belgium; Universidad de Sevilla, SPAIN

## Abstract

Monitoring the complex transmission dynamics of a bacterial virus (temperate phage P22) throughout a population of its host (*Salmonella* Typhimurium) at single cell resolution revealed the unexpected existence of a transiently immune subpopulation of host cells that emerged from peculiarities preceding the process of lysogenization. More specifically, an infection event ultimately leading to a lysogen first yielded a phage carrier cell harboring a polarly tethered P22 episome. Upon subsequent division, the daughter cell inheriting this episome became lysogenized by an integration event yielding a prophage, while the other daughter cell became P22-free. However, since the phage carrier cell was shown to overproduce immunity factors that are cytoplasmically inherited by the P22-free daughter cell and further passed down to its siblings, a transiently resistant subpopulation was generated that upon dilution of these immunity factors again became susceptible to P22 infection. The iterative emergence and infection of transiently resistant subpopulations suggests a new bet-hedging strategy by which viruses could manage to sustain both vertical and horizontal transmission routes throughout an infected population without compromising a stable co-existence with their host.

## Author Summary

Extensive co-evolution with their host has shaped bacterial viruses into the most abundant and sophisticated pathogens known to date. However, how these important viral pathogens manage to safely exploit their host without jeopardizing stable co-existence remains a central question, since horizontal (lytic) transmission can reduce the number of susceptible host cells and cause pathogen extinction, while vertical (lysogenic) transmission impairs pathogen abundance. Scrutinizing transmission of temperate phage P22 throughout a bacterial population at single cell resolution now revealed that this phage is able to disseminate immunity factors that allow the emergence of transiently resistant subpopulations of host cells. The continued fostering and consumption of such subpopulations points to an entirely new strategy by which viruses could manage to sustain an active infection with their host.

## Introduction

Viruses that infect microorganisms are ubiquitous in nature and often outnumber their hosts by an order of magnitude [1]. Their predatory behavior imposes a tremendous selective pressure able to affect host mutation rates [2], direct the global biogeochemical carbon flux [3] and structure microbial communities in many environments, including the gastrointestinal tract [3–5]. Furthermore, their gene transfer capacities and the ability of temperate viruses to integrate into the host chromosome are continuing to shape microbial genomes and adaptability [6–8].

The biology and life cycle of bacterial viruses (termed bacteriophages or phages) has been extensively studied and so far has revealed a plethora of phage–host interactions along the lines of two distinct reproductive strategies. In fact, it has long been established that upon infection the incoming phage chromosome can commit to replication and the production of new phage particles that are typically released by lysing the host and that enable further horizontal transmission [9]. During this lytic development, phage–host interactions are typically aimed at hijacking the host machinery and resources for massive replication of phage chromosomes and production of capsid proteins [10,11]. In case of temperate phages, the incoming phage chromosome can alternatively decide to lysogenize the host and persist as a dormant prophage that remains episomal or integrates in the host chromosome, where it becomes stably replicated and segregated, to ensure further vertical transmission [12,13]. In this lysogenic state, the genes supporting lytic development are typically repressed, with production of the corresponding phage-encoded repressor often being sustained by a toggle switch mechanism [14].

Despite these current paradigms of phage biology, however, the function and necessity of most phage encoded proteins still remains obscure while often the ecological complexity of phage-host associations remains unresolved [13,15,16]. In fact, how these important viral pathogens manage to safely exploit their host without jeopardizing stable co-existence remains a central question, since horizontal (or lytic) transmission can cause pathogen extinction by converting host cells to phage particles that suffer rapid physical decay in natural settings [17,18], while vertical (or lysogenic) transmission impairs pathogen virulence by converting host cells to superinfection resistant lysogens from which the dormant prophage can only rarely escape [19].

Our current lack of understanding might stem from the fact that most of the insights into phage biology are primarily derived from bulk level approaches that tend to overlook more subtle but nevertheless deterministic phage-host interactions that are either transient or operative in only a fraction of the infected population. The improvement of single-cell analysis approaches, however, is bringing such elusive insights within reach, and has started to shed light on the dynamic spatiotemporal regulation and orchestration of phage replication inside the cell [20–23].

In order to expand this view towards phage infected populations, we adopted a live cell biology approach to scrutinize the dynamic transmission of the P22 temperate phage throughout a population of its *Salmonella* Typhimurium host at single-cell resolution. As such, we found evidence for a mechanism that imposes the dissemination of phage-borne immunity factors to transiently protect emerging subpopulations of host cells from phage infection.

## Results

### Validation of the P22 chromosome tracking system in *S*. Typhimurium

**Fig 1:**
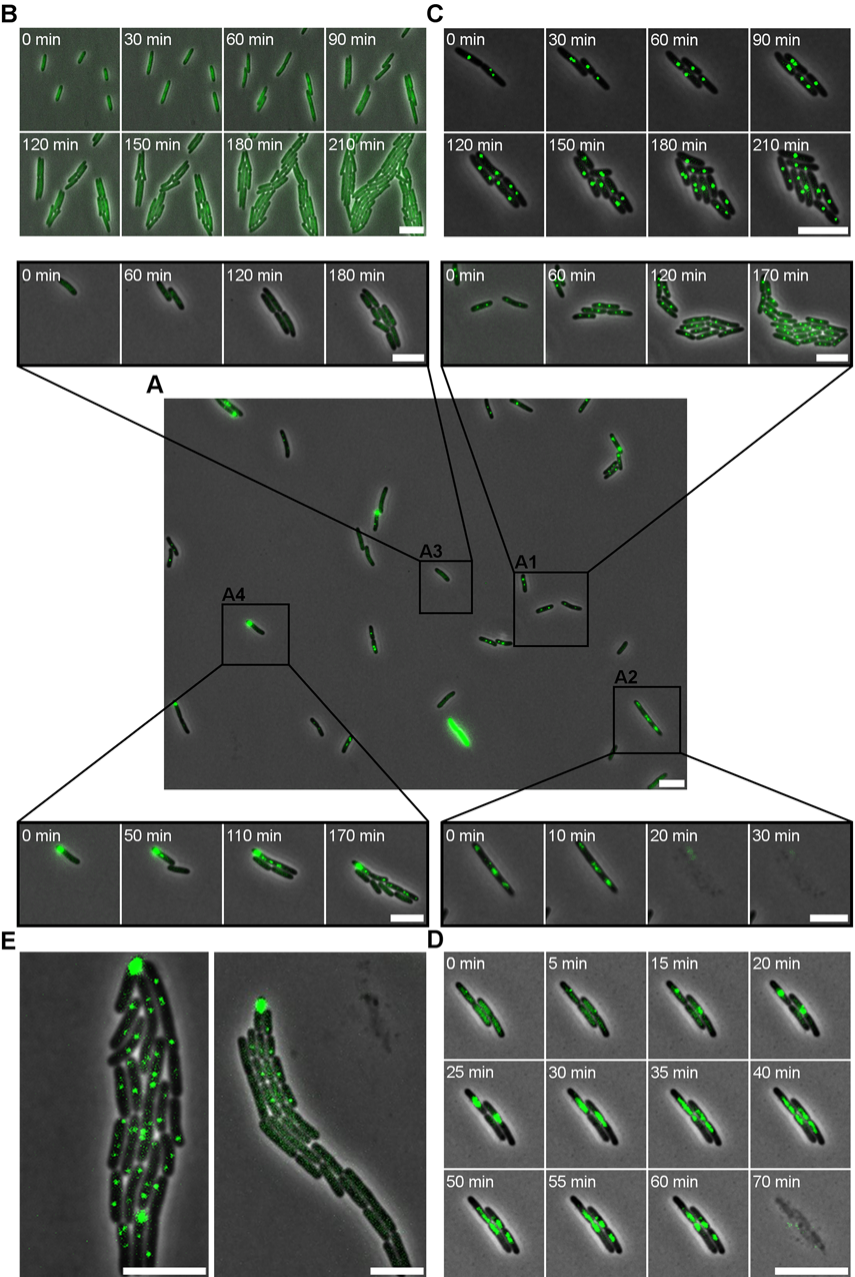
Categorizing single cell infection dynamics in the P22–*S*. Typhimurium model system. In the LT2ΔpSLT/pALA2705 reporter, presence of a P22 chromosome is revealed by the formation of a fluorescent GFP focus originating from GFP-ParB molecules bound to the *parS* sequence engineered in P22 *parS*. (A) The central picture shows an initial snapshot of an exponential phase population of LT2ΔpSLT/pALA2705 infected with P22 *parS* (MOI = 20) and further grown for 4 hours post infection in a semi-continuous culture. Time-lapse recordings of specific events are presented as a zoom in on the original snapshot as indicated by the black lines (box A1–A4). Four types of phage–host associations are seen in panel A: lysogenized cells in which the stably integrated P22 *parS* prophage yields a discrete GFP focus that replicates and segregates together with the host chromosome (box A1); lytically infected cells in which the replicating P22 *parS* chromosome yields a more diffuse and randomly dispersed GFP cloud throughout the cell prior to cell lysis (box A2); P22-free cells in which the absence of a P22 *parS* chromosome yields a diffuse cytoplasmic GFP fluorescence (box A3); phage carrier cells in which a polarly tethered P22 *parS* episome yields a coherent GFP cloud in one of the cell poles (box A4). Please note that the bright fluorescent cell at the bottom of panel A is a rare artifact. (B-D) Time-lapse series of (B) cells in the absence of P22 *parS*, (C) of growing cells from a P22 *parS* lysogen in LT2ΔpSLT/pALA2705, and (D) of LT2ΔpSLT/pALA2705 cells infected with P22 *c2 parS* (an obligate lytic derivative of P22 *parS*). (E) Snapshots from the lineages emerging from two phage carrier cells within a P22 *parS* infected LT2ΔpSLT/pALA2705 population, exhibiting either direct (left panel) or delayed (right panel) integration of the P22 *parS* prophage, resulting either in a homogeneous population of lysogens (left panel) or a heterogeneous population of both lysogens and P22-free cells (right panel). Analysis of 114 such lineages revealed the segregation of P22-free siblings in ca. 41% of cases. Phase contrast images (showing the cells) and GFP signal (reporting the P22 *parS* chromosomes) are merged. A 5 μm scale bar is shown at the bottom right of each panel. Timestamps are shown in the top left corners of time-lapse images. In panel D the timestamp is set at 0 min from the moment a ParB-GFP foci became visible. In all other panels the timestamp was started when first image was taken.

In order to be able to specifically track the intracellular whereabouts of the P22 chromosome during live infection of its *S*. Typhimurium LT2 host with time-lapse fluorescence microscopy, we (i) recombineered a *parS* sequence into the P22 chromosome (yielding P22 *parS*; [24]), and (ii) equipped its LT2 host with the pALA2705 plasmid ([25]; yielding LT2/pALA2705) expressing a cognate GFP-ParB fusion protein that binds to and multimerizes around this *parS* locus [26]. In the course of these experiments, however, we noticed that presence of the pSLT virulence plasmid in LT2 interfered with the proper localization of GFP-ParB (likely mediated by the presence of the pSLT specific *parS*/ParAB segregation system [27]; S1A Fig). Once the pSLT plasmid was cured from LT2 (resulting in LT2ΔpSLT/pALA2705), GFP-ParB assumed the proper diffuse cytoplasmic distribution indicative for the absence of a *parS* sequence in the cell (Figs 1 and S1B).

Upon further validation of this reporter system, a P22 *parS* lysogen of LT2ΔpSLT/pALA2705 was monitored with time-lapse fluorescence microscopy (Fig 1C). As anticipated, the corresponding cells stably carried one or two clearly delineated GFP-ParB foci in the middle of the cell, which upon subsequent cell divisions segregated symmetrically between daughter cells, as such reflecting lysogenic propagation of the P22 chromosome as a prophage that is integrated in and co-replicated with the host chromosome. Subsequently, the lytic mode of phage propagation was examined by monitoring the infection of LT2ΔpSLT/pALA2705 with an obligatory lytic P22 *c2 parS* (i.e. clear) mutant (Fig 1D). While the GFP-ParB labeled P22 chromosome appeared in the host cytoplasm within minutes after exposure to this phage, the corresponding GFP-ParB focus (i) gradually increased in size (in comparison with a single copy prophage genome shown in Fig 1C), indicative for P22 DNA replication, and (ii) eventually became less coherent to the point where it spread throughout the cytoplasm. Finally, as a last step before cell lysis, the GFP-ParB cloud shrunk, most likely indicating phage packaging and GFP-ParB release from the *parS* site in the P22 chromosome.

### Tracking the intracellular whereabouts of the P22 chromosome throughout an actively infected *S*. Typhimurium population reveals a P22-free and immune subpopulation

**Fig 2:**
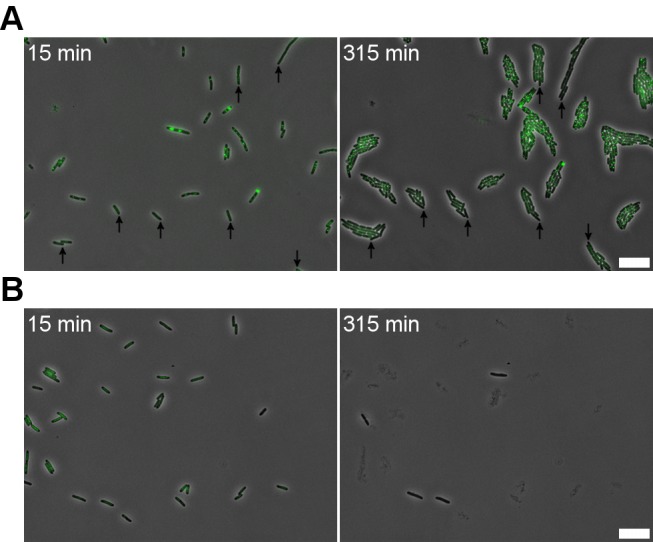
P22 infection can endow P22 immunity upon a subpopulation of P22-free host cells. (A) An exponential phase LT2ΔpSLT/pALA2705 population infected with P22 *parS* (MOI = 20) was grown for 4 hours in a semi-continuous culture after which an aliquot was mixed with P22 H5 (an obligate lytic mutant of P22; MOI = 20) and subjected to time-lapse microscopy. Arrows indicate phage free cells in the population (as recognized by their diffuse GFP-ParB fluorescence) remaining resistant to P22 H5 and P22 *parS* infection for over 300 minutes (left panel). Out of the 383 cells observed in this experiment, ca. 20% appeared to be P22-free and resistant. (B) An LT2ΔpSLT/pALA2705 control population grown in the same manner as in (A) but not previously exposed to P22 *parS*, either lysed or ceased growth when exposed to P22 H5 (MOI = 20). Phase contrast images (showing the cells) and GFP signal (reporting the P22 *parS* chromosomes) are merged. A 10 μm scale bar is shown at the bottom right of each panel. Timestamp in the upper left corner indicates time after mixing with P22 H5.

After having validated the whereabouts of the P22 chromosome during the two canonical modes of phage propagation, an LT2ΔpSLT/pALA2705 population was infected with P22 *parS* for four hours before the ongoing infection was monitored with time-lapse fluorescence microscopy (Fig 1A). Surprisingly, apart from cells undergoing lysogenic (Fig 1A1) and lytic (Fig 1A2) phage–host associations, a large number of P22-free cells could be observed whose integrity and growth appeared unaffected by the surrounding P22 *parS* phage particles (Fig 1A3). In fact, when the behavior of these P22-free cells was monitored on an agar-pad seeded with an obligate lytic mutant of P22 (i.e. P22 H5) to ensure a high surrounding concentration of predatory phage, this P22 immune growth could further be confirmed (Fig 2A). Since this behavior clearly differed from naïve LT2ΔpSLT/pALA2705 cells (i.e. not previously exposed to P22), which all became inactivated and typically lysed in the presence of P22 H5 (Fig 2B), these observations indicate that prior exposure to P22 infection can endow P22 immunity upon a subpopulation of host cells. The origin and physiology of this P22-free and immune subpopulation is further scrutinized below.

### P22-free cells can originate from the phage carrier state that precedes lysogenic conversion

In parallel to observing P22-free cells, cells supporting the phage carrier state of P22 were regularly observed in an active infection as well (Fig 1A4). This phage carrier state was most recently described by our group as a distinct polarly located episome of P22 DNA that upon subsequent cell divisions segregates asymmetrically between daughter cells, and that throughout several generations becomes inherited by only one of the siblings [24]. However, due to interference of the pSLT plasmid with proper GFP-ParB tracking (see above) it was previously impossible to scrutinize the peculiarities of the phage carrier state into more detail.

Most interestingly, it could now be observed that the lineage emerging from such a phage carrier cell could give rise to both P22-lysogenized (i.e. displaying coherent and nucleoid associated GFP-ParB foci) and P22-free (i.e. displaying a diffuse cytoplasmic GFP-ParB fluorescence) cells. In fact, closer examination of such emerging lineages revealed that often the first sister cell(s) of the phage carrier cell became P22-free, after which typically the phage carrier cell itself became lysogenized by an integration event and further gave rise to P22-lysogenic siblings (Fig 1E; right panel). This formation of P22-free siblings is actually in agreement with very early findings of Zinder [28], who observed the appearance of up to 60% non-lysogenized clones to emerge from a *S*. Typhimurium population undergoing lysogenic conversion by P22.

Importantly, throughout our experiments no integration events could be detected in the absence of a prior phage carrier state, indicating that this state invariably precedes prophage formation (even if no P22-free cells are formed; Fig 1E; left panel). Furthermore, the sustained presence of the polar GFP-ParB focus even after the integration event underscores that a number of P22 chromosomes (rather than a single copy) are polarly tethered in the phage carrier cell. The latter observation is in fact reminiscent with early biochemistry experiments suggesting the formation of a possibly membrane tethered replication complex before commitment to either lytic or lysogenic development of P22 [29–31].

### P22-free siblings from the phage carrier cell are immune to P22 infection

**Fig 3:**
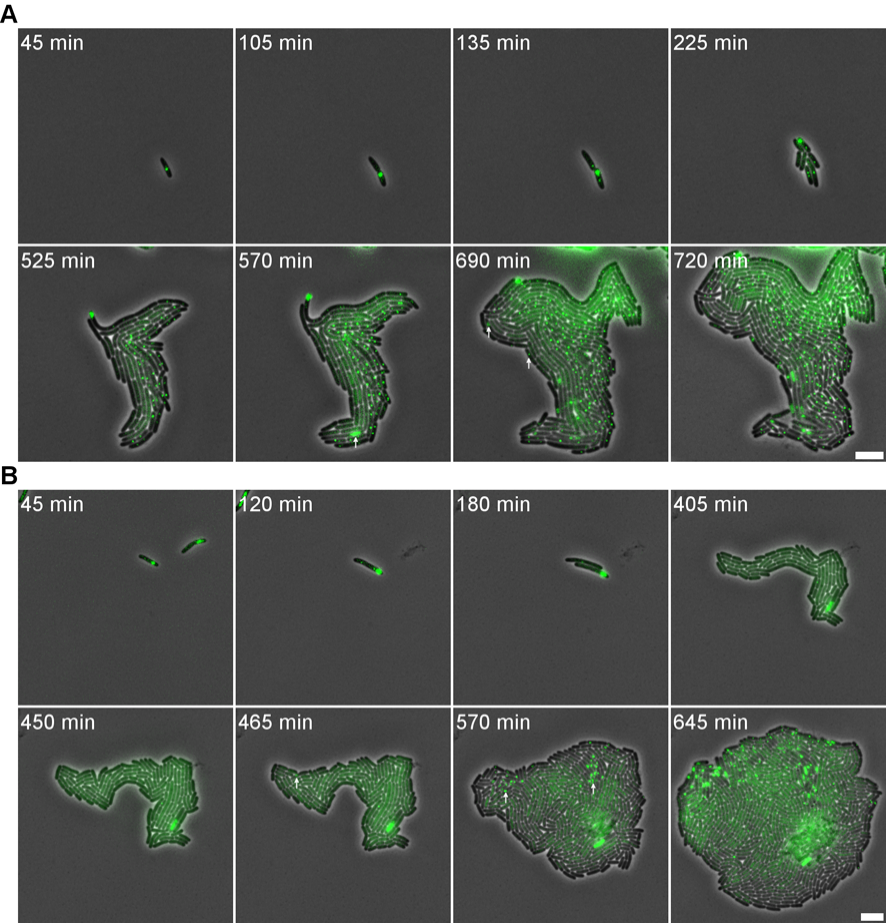
P22-free siblings segregated from a phage carrier cell are transiently immune to P22 infection. An exponential phase population of LT2ΔpSLT/pALA2705 was infected with (A) P22 *parS* or (B) P22 *Δint parS* (MOI = 5), and incubated for 15 minutes before plating on an agar pad seeded with additional P22 *parS*. P22-free siblings remained resistant to superinfection until time point (A) 525 min and (B) 450 min. For clarity some events of P22 *parS* infection are marked by a white arrow. Phase contrast images (showing the cells) and GFP signal (reporting the P22 *parS* chromosomes) are merged. A 5 μm scale bar is shown at the bottom right of each panel. Timestamps shown in the upper left corner of each frame indicate time after addition of the respective phages.

To further examine whether the peculiar population of P22-free but immune cells in Fig 1A3 did indeed stem from the phage free siblings of the phage carrier cells described above, the lineage stemming from a P22 *parS* phage carrier cell was monitored on an agar-pad seeded with P22 *parS* (Fig 3A; until 525 min). This clearly demonstrated that P22-free cells immune to P22 entry did indeed spawn off from the phage carrier cell prior to the actual lysogenization (i.e. integration) event of the latter. Furthermore, when a similar experiment was performed with a P22 Δ*int parS* mutant that is unable to integrate itself into the chromosome as a prophage (and thus unable to lysogenize the host cell), all phage carrier sister cells became P22-free and immune, indicating that the actual integration event was not essential to yield this immunity (Fig 3B; until 450 min). Most likely, this immunity is also the reason behind the peculiar observation that a P22 *Δint* mutant still gives rise to turbid plaques despite its inability to lysogenize the host.

In the above experiments, the occasional appearance of a single focus in a non-lysogenized sibling of the phage carrier cell (as observed in Fig 3B throughout the time-lapse recording) likely represents an un-integrated P22 chromosome that either detached from the tethered carrier state or managed to enter a P22-free immune cell. Since they do not commit to lytic infection, such single copy P22 chromosomes are likely repressed and toggled by the C2 repressor, therefore remaining idle (as will be proven and discussed further below).

### Immunity in P22-free siblings is transient and allows the emergence of a P22-susceptible subpopulation

Surprisingly, upon further monitoring the P22-free lineage spawning off a phage carrier cell, we observed that its immunity was transient (and thus not mutationally fixed), as after a number of generations the lineage eventually became infected by P22 *parS* (Fig 3A and 3B, last frames). Additionally, when a P22 *Δint parS* infected culture was monitored with time-lapse microscopy five hours after infection, we were able to observe phage propagation through a single, exponentially growing, P22-free lineage from the moment its transient resistance disappeared (S1 Movie). More specifically, the surrounding P22 *parS* phages quickly propagated throughout the whole colony, thereby forming new P22 particles, new phage carrier cells and a new population of transiently resistant P22-free cells (S1 Movie). Furthermore, upon maintaining a P22 *parS* infected population under continuous liquid culture for up to 20 host generations, transiently immune P22-free cells could still be observed within the well mixed population, indicating that even on a longer time frame this transient immunity supports a subpopulation of phage free siblings that eventually becomes susceptible for *de novo* P22 infection and as such sustains the co-existence of phages, prophages and susceptible hosts within the infected population.

### Transient immunity in P22-free siblings is derived from cytoplasmic inheritance and dilution of P22-borne immunity factors produced in the phage carrier cell

Interested in the molecular mechanism underlying this transient immunity of P22-free siblings, we noticed that the first sister cells spawning off a P22 Δ*int parS* phage carrier cell in general also seemed predisposed to be the first cells of the emerging lineage to lose their immunity and become infected, suggesting that an immunity determinant is originating from the phage carrier cell and cytoplasmically passed down and diluted to subsequent generations of P22-free siblings up to the point where it becomes insufficient to prevent infection.

In order to identify P22-borne determinants that could potentially participate in conferring immunity against P22 infection, a shotgun plasmid library of the P22 genome in *S*. Typhimurium LT2 was screened for clones resistant to P22 infection upon cross-streaking on LB agar plates. As such, the *gtrABC* (encoding proteins for LPS modification; [32,33]), *sieA* (encoding a superinfection exclusion factor; [34]) and *c2* (encoding the repressor of the lytic cycle; [12]) loci were each found to confer P22-resistance when individually expressed in a *S*. Typhimurium host. Moreover, these three factors have previously been described in literature to prevent superinfection of an established P22 lysogen by compromising (i) attachment of the P22 particle to the host LPS by modifying the O-antigen (via *gtrABC*), (ii) entry of the P22 chromosome through a currently unknown mechanism (via *sieA*) and (iii) its lytic proliferation (via *c2*) [12,32,34].

**Fig 4:**
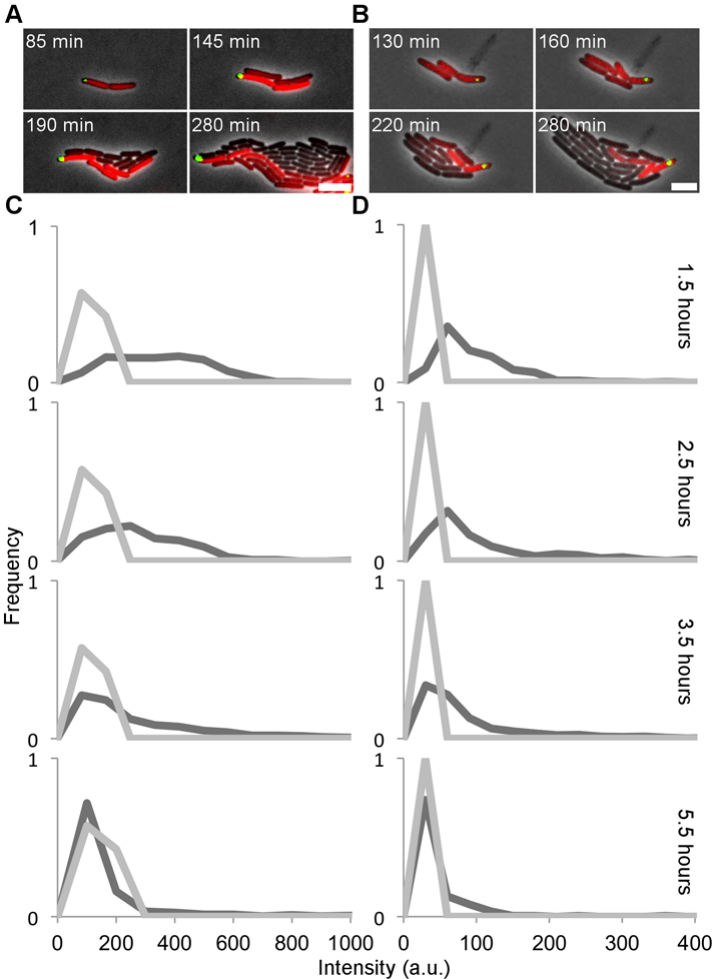
P22-borne expression of *sieA* and *gtrABC* is activated in the phage carrier state at high levels in comparison to the prophage state. (A, B) Infection of an exponential phase population of LT2ΔpSLT/pCW-*mCherry-parB* with (A) P22 *gtrABC-yfp parS* or (B) P22 *sieA-yfp parS* shows that *gtrABC* and *sieA* expression (shown in red), respectively, is linked to the phage carrier state (shown in green), after which the fluorescence is cytoplasmically inherited and diluted by its sister cells. A 5 μm scale bar is shown at the bottom right of each panel. Timestamps shown in the upper left corner of each frame indicate time after addition of the respective phages. (C, D) Quantitative analysis of single-cell YFP fluorescence intensities (expressed as arbitrary units; a.u.) of individual cells of LT2ΔpSLT/pCW-*mCherry-parB* in a semi continuous culture infected with (C) P22 *gtrABC-yfp parS* (MOI = 10) or (D) P22 *sieA-yfp parS* (MOI = 10) sampled at the indicated time points after initiation of infection (dark grey lines). For comparison, single-cell YFP fluorescence intensities of (C) P22 *gtrABC-yfp parS* and (D) P22 *sieA-yfp parS* lysogens in LT2ΔpSLT/pCW-*mCherry-parB*, after 4 hours of exponential growth are shown in each graph (light gray lines). Please note that YFP intensities of non-infected (i.e. negative control) cells averaged at 3.12 a.u. (average of 542 cells; Standard deviation = 2.21 a.u.). Every graph represents the analysis of between 134 and 461 single cells.

When P22-borne expression of *gtrABC* and *sieA* was made fluorescently tractable (via transcriptional fusions to the YFP fluorescent protein), however, it could be observed that the phage carrier cell expressed these factors to a much higher extent than established lysogens did (Fig 4). In fact, while it was recently reported that the P22 *gtrABC* promoter is subject to phase variable expression in a P22 lysogen [33], its expression seemed constitutively ON in the phage carrier state. Eventually, the fluorescent proteins produced from the *gtrABC* and *sieA* promoters in the phage carrier state became progressively diluted upon subsequent inheritance by the P22-free sister cell and its siblings (Fig 4), indicative for the vertical dilution of these immunity factors in the P22-free lineage.

**Fig 5:**
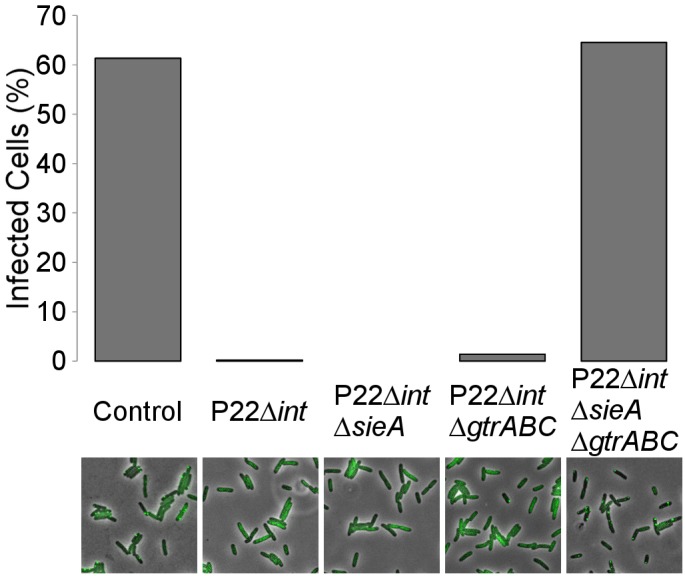
SieA or GtrABC independently account for P22 immunity in P22-free cells. An exponential phase population of LT2ΔpSLT/pALA2705 was infected with either no phage (control) or with the indicated phages (MOI = 10). After 4 hours of semi-continuous growth, cultures were plated on agar pads seeded with P22 *parS* and after 45 minutes the percentage of cells infected with this phage was scored. Percentages were obtained by analysis of between 508 and 646 single cells per experiment. Below each bar-plot, a representative snapshot of each respective experiment is shown in which phase contrast images (showing the cells) and GFP signal (reporting the P22 *parS* chromosomes) are merged.

To further confirm whether the cytoplasmic inheritance of these two factors indeed plays a role in the (transient) immunity of the P22-free cells, the phage carrier state was established with P22 *Δint* derivatives compromised in either or both *gtrABC* and *sieA* expression and the resulting immunity was assessed after four hours of growth. As expected from the expression during the phage carrier state (Fig 4) only phage carrier cells generated by a P22 derivative in which both *gtrABC* and *sieA* were deleted (i.e. P22 *Δint ΔgtrABC ΔsieA*) gave rise to P22-free siblings that could readily be infected with P22 *parS*, indicating that GtrABC and SieA factors both independently confer the observed immunity by preventing entry of the P22 chromosome in P22-free cells (Fig 5). Notably, the latter further underscores that *gtrABC* expression is invariably activated during the phage carrier state irrespective of the phase variability of the *gtrABC* promoter.

### GtrABC and SieA mediated immunity prevents premature silencing of the incoming P22 chromosome by the inherited C2 repressor

**Fig 6:**
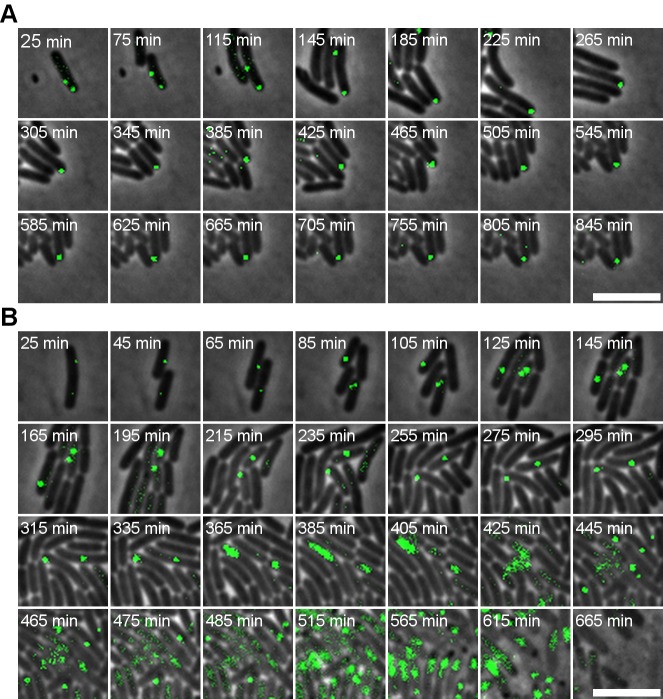
Loss of P22-borne *gtrABC* and *sieA* expression causes premature entering and C2-based silencing of incoming P22 chromosomes in P22-free cells. P22-free cells derived from an exponential phase population of LT2ΔpSLT/pALA2705 initially infected with P22 *Δint ΔgtrABC ΔsieA* (MOI = 10) and propagated semi-continuously for 4 hours, were mixed with P22 *parS* (A) and P22 *c2 parS* (B) (at MOI = 10), and monitored with time-lapse fluorescence microscopy. Phase contrast images (showing the cells) and GFP signal (reporting the incoming P22 *parS* chromosomes) are merged. A 5 μm scale bar is shown at the bottom right of each panel. Timestamps shown in the upper left corner of each frame indicate time after addition of the respective phages.

Interestingly, while in a P22 *Δint ΔsieA ΔgtrABC* infected population the phage carrier cell and its sister cells were deprived of their transient immunity and readily got infected by P22 *parS*, we observed this incoming P22 chromosome to remain idle in the cytoplasm. The fact that the infecting P22 could not commit to lytic or lysogenic proliferation likely indicated its premature repression or silencing by C2 repressor proteins that are most probably cytoplasmically inherited from the phage carrier cell as well. Moreover, the observation that this apparent silencing did not become alleviated after a number of generations (during which the originally inherited C2 proteins should have been diluted out) suggested that the incoming P22 chromosome (upon exposure to the cytoplasmically inherited C2 concentration) itself became genetically toggled to sustained C2 repressor production (Fig 6A). In fact, the above assumption could be corroborated by replacing the incoming P22 *parS* phage with a P22 *c2 parS* derivative, which cannot be toggled to take over C2 production. The latter derivative indeed only transiently became silenced by the cytoplasmically inherited and further diluted C2 protein, after which it engaged in lytic infection (Fig 6B).

Moreover, in line with this ability of P22 *c2* mutants to overcome premature silencing and lytically take advantage of immunity-compromised P22-free siblings, we observed that during prolonged semi-continuous incubation (up to 30 host generations) of P22 *Δint ΔgtrABC ΔsieA* with LT2, cultures typically got overtaken by a phage population harboring *c2* mutations. Such dynamics were not observed with P22 *Δint*, indicating that the SieA and GtrABC mediated delay of P22-free cell infection abolishes the competitive advantage and thus enrichment of such *c2* mutants.

Finally, it should be noted that premature entry and subsequent silencing of P22 chromosomes was sporadically observed in (GtrABC and SieA proficient) P22-free immune cells generated during P22 *Δint parS* infection as well, indicating that GtrABC and SieA mediated prevention of this phenomenon is not absolute.

## Discussion

In order to scrutinize the transmission dynamics of a temperate bacterial virus (P22) throughout a population of host (*S*. Typhimurium) cells, we used live cell biology to track the different possible fates and whereabouts of individual host cells and phage chromosomes during an active infection. Aside host cells engaged in horizontal (lytic) and vertical (lysogenic) P22 transmission (Fig 1C and 1D), we surprisingly also observed P22-free but nevertheless transiently immune lineages to emerge from cells undergoing lysogenic conversion (Figs 2 and 3). More specifically, prior to its integration into the host chromosome, the incoming P22 chromosome was shown to first establish a polarly tethered P22 episome (referred to as the phage carrier state) that upon further divisions of the host cell (referred to as phage carrier cell) did not segregate to the daughter cell inheriting the other pole (Figs 1E, 3 and 4). Since the first division(s) of the phage carrier cell often preceded the actual integration event, P22-free siblings could emerge (prior to the establishment of lysogeny in the phage carrier cell) that nevertheless cytoplasmically inherited the immunity factors (i.e. GtrABC, SieA and C2) that were shown to be constitutively produced by P22 in the phage carrier state (Fig 4). In turn, these factors became vertically transmitted and diluted throughout the lineage emerging from the P22-free cells up to the point where the immunity became insufficient to further protect this lineage from P22 infection (Figs 3 and 6). Importantly, while the immunity factors transiently alleviated P22 susceptibility and therefore permitted proliferation of the P22-free subpopulation, GtrABC and SieA in particular also prevented the premature entry of P22 chromosomes (Fig 5) that would otherwise have become silenced by the residual C2 repressor cytoplasmically inherited from the phage carrier cell as well (Fig 6). As such, the imposed delay in P22 susceptibility allowed both the emergence and priming of a P22-free subpopulation for proper *de novo* P22 infection.

It might furthermore be anticipated that the gradual fading of the protective measures (such as LPS modification) imposed by cytoplasmic dilution of GtrABC and SieA activity in transiently immune P22-free siblings would at first liberate only a limited number of P22 attachment or entry sites on the cell surface, so that even at a high multiplicity of infection the majority of freshly susceptible cells would tend to undergo lytic infection (contributing to phage particle production) initiated by the entry of a single phage chromosome rather than lysogenic conversion enforced by the simultaneous entry of multiple phage chromosomes.

Although future research should proceed to address the exact molecular details of phage carrier state establishment and its prevalence in other phage-host systems, the iterative fostering and subsequent consumption of transiently immune subpopulations as revealed in this study could allow for a phage to sustain an active infection, and the concomitant production of phage particles, without compromising the co-existence with its host. Since horizontal and vertical phage transmission in principle tend either to deplete the population of susceptible host cells or to enrich the population in immune lysogens carrying the dormant prophage, respectively, the carrier state mediated supply of new susceptible host cells might constitute a bet-hedging mechanism that could adjust these dynamics.

In fact, virulent (i.e. strictly lytic) phages face a serious threat of extinction in the absence of fresh susceptible hosts [17,18], and several mechanisms accounting for the apparently stable co-existence of such phages with their hosts in nature have been forwarded. While structured populations such as biofilms were shown to harbor physically shielded refuges to secure the supply of susceptible bacteria [35,36], *in silico* models for homogenously mixed infected populations have since long proposed transient forms of phenotypic phage resistance to greatly stabilize phage-host coexistence [37]. Furthermore, several virulent phages were once suggested to have the ability to reside in their hosts and postpone lysis in order to allow their carrier cell to segregate new phage-free siblings [38–40]. These latter projections seem to be supported by our *in vivo* observations of a phage carrier state mediating such transient phenotypic phage resistance, although it remains to be established whether virulent phages could indeed embark in such a carrier state association. For temperate phages on the other hand, the carrier state mediated supply of new susceptible host cells could counterbalance the inevitable enrichment of prophage carrying lysogens with the production of free phage particles, in turn allowing proper bet-hedging between both repositories of the phage chromosome.

Our work also more generally draws attention to the presence and importance of more subtle or transient phage-host interactions that defy the traditional bifurcation of phage biology into lytic or lysogenic development, and that might draw on dedicated phage functions that account for part of the currently cryptic phage gene pool. As such, we could demonstrate that the phage carrier state expresses immunity factors previously thought to serve only superinfection exclusion of established lysogens. Moreover, we have previously found the cryptic P22-endoded *pid* gene to be particularly expressed in the carrier state [24]. Furthermore, in the wake of the polarly tethered phage carrier state and its impact on P22 transmission dynamics, we also revealed the sporadic appearance of a second carrier state, in which a P22 chromosome prematurely entering a P22-free cell still containing sufficient cytoplasmically inherited C2 repressor becomes silenced and toggled to sustain the C2 producing state. As such, this single and silenced P22 chromosome that seemingly remains idle in the cytoplasm deviates from a lytic, lysogenic and even polarly tethered P22 episome, but might nevertheless serve a biological purpose associated with dedicated phage genes as well. From another perspective, it is tempting to speculate that such non-lethal carrier state associations, by introducing an unintegrated idle phage chromosome in the host cytoplasm, could potentially provide the correct timing and substrate for the host cell to perhaps evolve and/or engage in CRISPR-Cas-like interactions [41] with the phage chromosome.

In summary, by scrutinizing viral transmission dynamics throughout a bacterial population at single cell resolution, this study revealed that P22 is able to disseminate immunity factors that allow the emergence of transiently resistant subpopulations of its *S*. Typhimurium host. The continued fostering and consumption of such subpopulations points to a new and population-level exploitation or farming strategy by which viruses could manage to sustain an active infection with their host.

## Materials and Methods

### Bacterial strains, phages and media

Bacterial strains, phages and plasmids constructed and used throughout this study are listed in Table 1. For general cloning and microbiology procedures, Lysogeny Broth (LB; [42]) was used either as a broth or as agar plates after the addition of 1.5% agar. When appropriate, the following chemicals (Applichem, Darmstadt, Germany) were added to the growth medium at the indicated final concentrations: ampicillin (100 μg/ml; Ap^100^), chloramphenicol (30 μg/ml; Cm^30^), kanamycin (50 μg/ml; Km^50^) and tetracycline (20 μg/ml; Tc^20^).

**Table 1:** Strains, phages and plasmids used in this study.

**Strains**		
LT2	*Salmonella* Typhimurium LT2 wild-type	[46]
LT2ΔpSLT	LT2 cured from the pSLT virulence plasmid	[47]
TH6939	High efficiency recombination strain containing pKD46	Diarmaid Hughes, Uppsala University, Sweden
TH8127	High efficiency recombination strain containing pSIM5	Diarmaid Hughes, Uppsala University, Sweden
T-SACK	Used as template for PCR amplification of the *tetA-sacB* counter-selection cassette for recombineering	[48]
**Phages**		
P22	Enterobacteria phage P22	SGSCa
P22 *parS*	*parS* inserted between gene *gtrC* and *9*	This study and [24]
P22 H5	Virulent derivative of P22	Kelly Hughes (University of Utah, USA)
P22 *c2 parS*	Clear variant of P22 *parS*, by engineering two stop codons after the first 11 codons of *c2*	This study
P22 *Δint parS*	P22 *parS* lacking the *int* gene and thus unable to integrate in its host’s chromosome	This study
P22 *sieA-yfp parS*	Transcriptional *yfp* fusion to *sieA* in a P22 *parS* background	This study
P22 *gtrABC-yfp parS*	Transcriptional *yfp* fusion to *gtrC* in a P22 *parS* background	This study
P22 *Δint*	P22 lacking the *int* gene and thus unable to integrate in its host’s chromosome	This study
P22 *Δint ΔsieA*	P22 *Δint* deleted of the *sieA* gene	This study
P22 *Δint ΔgtrABC*	P22 *Δint* deleted of the *gtrABC* operon	This study
P22 *Δint ΔsieA ΔgtrABC*	P22 *Δint* deleted of the *sieA* gene and the *gtrABC* operon	This study
**Plasmids**		
pALA2705	Encodes GFP-ParB under control of the *lac* promoter	[25]
pCW-*mCherry-parB*	Encodes mCherry-ParB under control of the *lac* promoter. Derived from pALA2705	[24]
pKD46	Encodes ʎ *red* genes under control of an arabinose inducible promoter	[49]
pKD13	Used as template for PCR amplification of a *frt*-flanked kanamycin resistance cassette for recombineering	[49]
pSIM5	Encodes ʎ *red* genes under control of a temperature inducible promoter	[50]
pCP20	Encodes Flp for recombining *frt* sites	[51]
pGBKD3-*parS*	Used as template for PCR amplification of the *parS* site together with a *frt-*flanked chloramphenicol resistance cassette for recombineering	[52]

Strains for microscopic analysis were typically grown overnight at 30°C in AB-glycerol medium (AB). Mininal AB medium contains 2 g/l (NH_4_)_2_SO_4_, 6 g/l Na_2_HPO_4_, 3 g/l KH_2_PO_4_, 3 g/l NaCl, 0.1 mM CaCl_2_, 1.0 mM MgCl_2_, 0.003 mM FeCl_3_ [43] and was supplemented with 0.2% D-glycerol, 1 μg/ml uracil and 1 μg/ml thiamine and 0.2% cas-amino acids. The following morning cultures were diluted 1:100 and grown for an additional 3–5 hours to reach an OD_100_ between 0.2 and 0.3 before commencing phage infection and microscopy analysis.

Phages were propagated on *S*. Typhimurium LT2 as plaques in LB soft-agar (0.7% agar) or as lysates in LB as described previously [44]. Phage stocks were filter sterilized with 0.2 μm filters (Fisher Scientific, Aalst, Belgium). To avoid carryover of nutrient rich LB to the microscopy pads or cultures with AB, phage suspensions used for microscopy analysis were PEG-precipitated according to [45]. Briefly, 400 μl of phage suspension was mixed with 400 μl 24% PEG-6000 and 50 mM NaCl, incubated 1 hour at 4°C, phages were pelleted by centrifugation at 8000 × *g* for 10 min and resuspended in 400 μl AB. Phage titers before and after PEG-precipitation were essentially similar.

Phage infected liquid cultures were grown in a semi-continuous batch system to maintain exponential growth. To achieve this, bacterial cultures were diluted 1:2 every hour to keep cell counts constant (i.e. doubling time in AB is approximately 1 hour).

### Construction of bacterial and phage mutants

For more efficient recombination, strains with increased transformation efficiency TH6939 (containing pKD46) and TH8127 (containing pSIM5) were used when appropriate (kind gift of Diarmaid Hughes, Uppsala University, Sweden). Because of the temperate nature of P22, mutations were typically constructed in the P22 prophage in the appropriate recombineering strain, after which the P22 mutants were collected from the supernatants of the corresponding lysogen. In this context, it should be noted that when more than two *frt* sites are present in the same P22 chromosome, the high efficiency of pCP20 mediated flipping tends to recombine all present *frt* sites in a random order, leading to unviable P22 prophages. Therefore, in these occasions pCP20 was not used, but whenever possible we used the endogenous tendency of P22 to recombine two close *frt* sites in larger than wild type P22 chromosomes, which was recently described by our group [53].

The *S*. Typhimurium LT2 strain lacking the pSLT plasmid was kindly provided by Josep Casadesús (Department of Genetics, Universidad de Sevilla, Spain; [47]) and transformed with pALA2705 [25] leading to LT2ΔpSLT/pALA2705. For the construction of P22 *parS*, the *parS-frt-cat-frt* cassette was PCR amplified from pGBKD3-*parS* [52] with primers P22_parS_Fw and P22_parS_Rev (Table 2), and inserted between the *gtrC* and *9* genes in LT2 lysogenized with P22 via recombineering [49], as described in [24]. Please note that no significant difference in plaque count or morphology was observed when plating P22 or P22 *parS* on either wild type LT2 or LT2ΔpSLT/pALA2705.

**Table 2:** Primers used in this study.

Primer name	Sequence (5’–3’)^a^
P22_parS_Fw	GAATATTTAACATAAAATAAAAATGGGTGTTTACACCCATTTTTATTACA **GATTGTGTAGGCTGGAGCTGC**
P22_parS_Rev	AAAAACCCAATGGAGAATTAGTTAGATTAACCTTGGCAACACTTTAGATA **GGTCTGCTATGTGGTGCTATCT**
Oligo_c2_Stop	ATGAATACACAATTGATGGGTGAGCGTATTCGC TAATAA GCTCGAAGAAAAAAACTCAAGATTAGACAAG
P22_Δint_Oligo	CAAAAGGCAGGCCACAGAGCTTCATGACAAGCTGAGTATTTGATTTAACTGGTGCCGATAATAGGAGTCG
P22_Δint_Fw	AAGGTCGTAGGTTCGACTCCTATTATCGGCACCAGTTAAATCAAATACTTA **AGGAACACTTAACGGCTGACAT**
P22_Δint_Rev	GCATACTGTCCAGGTGAGCGCGGGTGATGACATAACAGAGGAACTGAAATG **GTGTAGGCTGGAGCTGCTTC**
P22_ΔsieA_Fw	CAATGTTAACTTTTTTCATGGTATCCTGCACAAAACTAAGGAGGTTGGTGTG **ATTCCGGGGATCCGTCGACC**
P22_ΔsieA_Rev	TAGCTAACGAAGCTACCCGGCAGTAGCGCTATAAGCCAAGGACGGCATTTA **TGTAGGCTGGAGCTGCTTCG**
P22_ΔgtrABC_Fw	TTTGTAGTGCTACACTTCAGACCTTTCCGAATCCGCTGATTTTCATAATG **ATTCCGGGGATCCGTCGACC**
P22_ΔgtrABC_Rev	AAATGGGTGTAAACACCCATTTTTATTTTATGTTAAATATTCTATAGCTA **TGTAGGCTGGAGCTGCTTCG**
CW_ΔgtrABC_TetSac_Fw	TTGAAGTTATTCGCTAAGTACACATCGATCGGTGTTCTTAACACGCTTAT **TCCTAATTTTTGTTGACACTCTATC**
CW_ΔgtrABC_TetSac_Rev	ATTAAACCTAACAACTATGGTTTCCCCTACAACACCAATATCGTATACGT **ATCAAAGGGAAAACTGTCCATATGC**
P22_ΔgtrABC_Control_Fw	GATAGCGGAGCATTGTACTCCCAC
P22_ΔgtrABC_Control_Rev	CGCTCACACGTCCCATCTTC
P22_sieA_yfp_Fw	CCGGAGAAGCGAATGGATTTAATAAAGAATTCATAAAAACTATAAAATAA **TAAGAAGGAGATATACATATGGC**
P22_sieA_yfp_Rev	TTTAGCTAACGAAGCTACCCGGCAGTAGCGCTATAAGCCAAGGACGGCAT **TCCTCCTTAGTTCCTATTCC**
P22_gtrC_yfp_Fw	GTATACGATATTGGTGTTGTAGGGGAAACCATAGTTGTTAGGTTTAATTAG **TAAGAAGGAGATATACATATGGC**
P22_gtrC_yfp_Rev	ATAAAAATGGGTGTAAACACCCATTTTTATTTTATGTTAAATATTCTATAG **TCCTCCTTAGTTCCTATTCC**

^a^ When relevant, primer attachment sites are indicated in bold. Recombination regions in regular font and the two stop-codons introduced in *c2* are underlined

Clear mutant P22 *c2 parS* was constructed by oligo-mediated mutagenesis [54] of the P22 *parS* lysogen, using olignucleotide Oligo_c2_Stop (Table 2). This oligo introduced two flanking stop codons after the first 11 amino acids of the P22 C2 repressor. After recombination, transformants were inoculated in LB with wild type LT2 and grown overnight at 37°C to amplify the corresponding clear mutants. Afterwards, P22 clear mutants, were isolated by plaquing on LT2, and the *c2* mutation was verified by sequencing by Macrogen Europe (Amsterdam, The Netherlands).

P22 *Δint parS* was constructed by oligo-mediated mutagenesis [54] of the corresponding P22 *parS* lysogen, using P22_Δint_Oligo (Table 2). All other *Δint* mutants in P22 were constructed by recombineering [49] a PCR amplicon (Phusion DNA polymerase; Fermentas) from plasmid pKD13, using primer pair P22_Δint_Fw and P22_Δint_Rev (Table 2). Please note that without Int, excision of the prophage is not possible [55]. However, the late structural and lytic genes can still be expressed irrespective of the prophage being excised or not [56]. After induction with mitomycin C it was therefore feasible to obtain a phage particle that was able to package a full P22 *Δint* prophage out of the host’s genome due to the inherent promiscuous packaging by P22 [57]. The resulting phages produced normal turbid plaques and were unable to form stable lysogens on LT2. Please note that due to the inability of P22 *Δint* mutants to form lysogens, the *Δint* mutation was always introduced as the last step when constructing P22 genomes with multiple gene deletions or insertions that needed pCP20 independent removal of antibiotic cassettes (see above).

Superinfection exclusion mutants of P22 of *sieA* and *gtrABC* were constructed by recombineering [49] using pKD13 as the template for PCR amplifying the kanamycin cassette with respective primer pairs P22_ΔsieA_Fw and P22_ΔsieA_Rev, P22_ΔgtrABC_Fw and P22_ΔgtrABC_Rev (Table 2).

In the construction of P22 *Δint ΔsieA ΔgtrABC*, the efficiency of spontaneous removal of the antibiotic resistance cassette from *gtrABC* proved to be too low to detect [53]. Therefore, the *gtrABC* operon in a P22 *ΔsieA* lysogen was first replaced by the *tetA-sacB* PCR amplified from the *E*. *coli* strain T-SACK (kind gift of Lynn C. Thomason of the Court-lab; [48]) with primers P22_ΔgtrABC_TetSac_Fw and P22_ΔgtrABC_TetSac_Rev (Table 2). A subsequent recombineering step, replacing the *tetA-sacB* cassette with the Δ*gtrABC*::*frt* PCR product obtained with primers P22_ΔgtrABC_Control_Fw and P22_ΔgtrABC_Control_Rev (Table 2) derived from the pCP20 flipped P22 *ΔgtrABC*::*frt* lysogen, was obtained by counter selection against both the presence of *tetA* and *sacB* [48], leading to P22 *ΔsieA ΔgtrABC*, that was thereafter deleted for *int*, resulting in P22 *Δint ΔsieA ΔgtrABC*.

Transcripitonal *yfp* fusions to *sieA* and *gtrABC* in P22 *parS* were constructed by recombineering [49]. PCR products for the fusions were PCR amplified (Phusion DNA polymerase; Fermentas) from a genomic template harboring *yfp-frt-cat-frt* with respective primer pairs P22_sieA_yfp_Fw and P22_sieA_yfp_Rev, P22_gtrC_yfp_Fw and P22_gtrC_yfp_Rev (Table 2), resulting in P22 *sieA-yfp parS* and P22 *gtrC-yfp parS*.

Please note that all deletion and insertion mutants made by recombining dsDNA products gave a single *frt* scar after removal of the antibiotic cassette. In case of some P22 *Δint* mutants not constructed with oligo-mutagenesis a kanamycin cassette remained. All constructed mutants were confirmed by PCR with primer pairs annealing just outside of the region where homologous recombination took place.

### Fluorescence microscopy

Fluorescence microscopy and time-lapse fluorescence microscopy were performed with a temperature controlled (Okolab Ottaviano, Italy) Ti-Eclipse inverted microscope (Nikon, Champigny-sur-Marne, France) equipped with a TI-CT-E motorised condensor, a YFP filter (Ex 500/24, DM 520, Em 542/27), an mCherry filter (Ex 562/40, Dm 593, Em 641/75), a GFP filter (Ex 472/30, Dm 495, Em 520/35) and a CoolSnap HQ2 FireWire CCD-camera. For imaging, cells were placed between AB agar pads and a cover glass using Gene Frames (Life Technologies), essentially as described previously (Stewart et al. 2005), and incubated at 30°C. Images were acquired using NIS-Elements (Nikon) and resulting pictures were further handled with open source software FIJI (i.e. ImageJ; http://fiji.sc/Fiji). For the analysis of the GtrC-YFP and SieA-YFP expression levels, aliquots were taken from the appropriate cultures at indicated time points. YFP fluorescence intensities (arbitrary units) of single-cells were measured using the NIS-Elements (Nikon) software, corrected for background fluorescence and subsequently binned to create the fluorescence intensity distributions.

### Screening a P22 shotgun library for P22-borne immunity factors

A P22 shotgun library created in plasmid pFPV-P_BAD_-*gfp* (i.e. by replacing the *gfp* gene which is under control of the inducible arabinose promoter; [24]) was transformed by electroporation into competent LT2 and plated on LB Ap^100^ containing 0.02% Glucose and incubated overnight at 37°C. Single transformants were cross-streaked against P22 H5 on plates containing LB Ap^100^ and 0.02% arabinose. These plates were incubated for 5–6 hours to screen for plasmids able to confer immunity to P22 infection in LT2. Immune colonies were picked, purified and confirmed once more by cross-streaking across P22 H5 on fresh plates containing LB Ap^100^ and 0.02% arabinose. To confirm that the immunity was truly plasmid-borne (and not resistance stemming from mutations in the LT2 chromosome), the plasmids from resistant clones were extracted and again transformed to competent LT2 to confirm the resistance phenotype. Sequencings of relevant inserts were performed by Macrogen Europe (Amsterdam, The Netherlands).

## Supporting Information

S1 Fig Influence of pSLT on the aspecific binding of GFP-ParB in *Salmonella* Typhimurium LT2. (A) LT2/pALA2705 showing discrete foci even in the absence of the phage P1 *parS* sequence. (B) Removal of pSLT from LT2 (resulting in LT2ΔpSLT/pALA2705) results in cells with a diffuse GFP-ParB distribution indicating the loss of aspecific binding sites for GFP-ParB. A 5 μm scale bar is shown at the bottom right of each panel.
(TIF)

S1 Movie Time-lapse movie showing phage propagation throughout a single, exponentially growing, P22-free lineage from the moment its transient resistance disappears. An exponential phase population of LT2ΔpSLT/pALA2705 was infected with P22 *Δint parS* (MOI = 0.1) and grown in a semi-continuous culture for 5 hours before plating on an agar pad seeded with additional P22 *parS*. Please note that phage entry can occur with either P22 *Δint parS* already present in the culture or the added P22 *parS*. Analysis of 33 similar lineages revealed that under these conditions transient resistance on average lasted for ca. 12 generations, based on a doubling time of ca. 55 min in AB-glycerol media. Phase contrast images (showing the cells) and GFP signal (reporting the P22 *parS* chromosomes) are merged. A 5 μm scale bar is shown in the bottom right and the timestamp indicates time after plating cells for microscopy analysis.
(AVI)
