# Supplementary figures and images for: Viral Transmission Dynamics at Single-Cell Resolution Reveal Transiently Immune Subpopulations Caused by a Carrier State Association
Source: PLoS Genet. 2015 Dec 31;11(12):e1005770. doi: 10.1371/journal.pgen.1005770 (PMC4697819; doi:10.1371/journal.pgen.1005770)

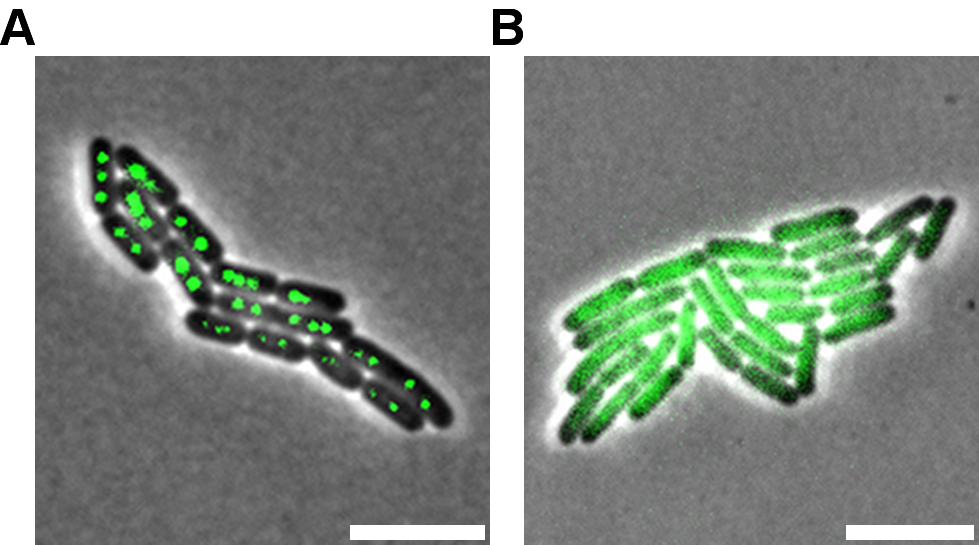

Supplement: S1 Fig — (A) LT2/pALA2705 showing discrete foci even in the absence of the phage P1 parS sequence. (B) Removal of pSLT from LT2 (resulting in LT2ΔpSLT/pALA2705) results in cells with a diffuse GFP-ParB distribution indicating the loss of aspecific binding sites for GFP-ParB. A 5 μm scale bar is shown at the bottom right of each panel. (TIF) [file pgen.1005770.s001.tif]
